# Canine Bone Marrow Mesenchymal Stem Cell Conditioned Media Affect Bacterial Growth, Biofilm-Associated *Staphylococcus aureus* and AHL-Dependent Quorum Sensing

**DOI:** 10.3390/microorganisms8101478

**Published:** 2020-09-26

**Authors:** Dobroslava Bujňáková, Anna Čuvalová, Milan Čížek, Filip Humenik, Michel Salzet, Daša Čížková

**Affiliations:** 1 Institute of Animal Physiology, Centre of Biosciences of the Slovak Academy of Sciences, 040 01 Košice, Slovakia; cuvalova@saske.sk; 2 Center for Experimental and Clinical Regenerative Medicine, University of Veterinary Medicine and Pharmacy in Košice, 040 01 Košice, Slovakia; milan.cizek@uvlf.sk (M.Č.); humenik@uvlf.sk (F.H.); dasa.cizkova@uvlf.sk (D.Č.); 3 Université Lille, Inserm, CHRU Lille, U-1192-Laboratoire Protéomique, Réponse Inflammatoire et Spectrométrie de Masse-PRISM, F-59000 Lille, France; michel.salzet@univ-lille.fr; 4 Institut Universitaire de France, 75000 Paris, France

**Keywords:** quorum sensing, biofilm, *Escherichia coli* JM109 pSB1142, mesenchymal stem cells

## Abstract

The present study investigated the in vitro antibacterial, antibiofilm and anti-Quorum Sensing (anti-QS) activities of canine bone marrow mesenchymal stem cell-conditioned media (cBM MSC CM) containing all secreted factors <30 K, using a disc diffusion test (DDT), spectrophotometric Crystal Violet Assay (SCVA) and Bioluminescence Assay (BA) with QS-reporter *Escherichia coli* JM109 pSB1142. The results show a sample-specific bacterial growth inhibition (zones varied between 7–30 mm), statistically significant modulation of biofilm-associated *Staphylococcus aureus* and *Escherichia coli* bioluminescence (0.391 ± 0.062 in the positive control to the lowest 0.150 ± 0.096 in the experimental group, cf. 11,714 ± 1362 to 7753 ± 700, given as average values of absorbance A_550_ ± SD versus average values of relative light units to growth RLU/A_550_ ± SD). The proteomic analysis performed in our previous experiment revealed the presence of several substances with documented antibacterial, antibiofilm and immunomodulatory properties (namely, apolipoprotein B and D; amyloid-β peptide; cathepsin B; protein S100-A4, galectin 3, *CLEC3A*, granulin, transferrin). This study highlights that cBM MSC CM may represent an important new approach to managing biofilm-associated and QS signal molecule-dependent bacterial infections. To the best of our knowledge, there is no previous documentation of canine BM MSC CM associated with in vitro antibiofilm and anti-QS activity.

## 1. Introduction

As antibiotic resistance has increased rapidly, thereby greatly limiting the medications available to treat chronic bacterial infections in clinical practice, the development of a new antimicrobial agent, especially one effective against multidrug resistant pathogens [1] and/or bacteria living in biofilm with adaptation resistance, is an urgent issue [2].

As multipotent adult stem cells with the capacity to differentiate into multiple cell types [3,4] and with a paracrine function, mesenchymal stem cells (MSCs) may be one of the options offering new prospects for the prevention and treatment of infections, for limiting the selection and spread of antibiotic resistance, or for potentially acting as drug delivery vehicles and enhancing the effectiveness of conventional antimicrobials. Recent data show that MSCs exert strong antimicrobial effects through indirect and direct mechanisms, partially mediated by the secretion of antimicrobial peptides and proteins (AMPs) [1,5,6,7,8]. Unlike human and mouse MSC counterparts, little is known about the characterization and function of canine MSCs, and this knowledge gap impedes the development of canine evidence-based MSC technologies [9].

Interest in mesenchymal stem cells (MSCs) for regenerative and reparative therapies, both in human and animals, is emerging, as the current treatment options for several conditions often do not result in either the desired clinical outcome or the patients’ return to normal function [10]. Canine MSCs have been evaluated in some experimental and preclinical studies on the efficacy and safety testing of novel treatments for humans, since dogs are considered as potential animal models for human disease research (based on several reasons, such as a relatively long life span, large body mass and natural disease onset) [11] and have a prevalent role in the development of new therapeutic cell-based approaches. Many human immune-mediated diseases leading to inflammatory processes have their canine homolog (such as canine atopic dermatitis, chronic gingivostomatitis, inflammatory bowel disease and asthma, among others) [12]. In addition, dogs share many similar pathologies with humans; they represent a perfect model for human conditions, a much better one than artificially created diseases in laboratory animals. Thus, research on canine-derived MSCs may provide insight into stem cell therapy not just for canines, but for humans as well [13].

Although these MSCs can be derived from several sources, clinical use has favored bone marrow and adipose tissue because of their relative ease of stem cell recovery in large numbers and their minimal donor-site morbidity [10]. Despite the fact that adipocyte-derived MSCs have a higher ability of proliferation and are more readily available, BM MSCs showed a higher production of paracrine factors [14,15].

Fibroblasts may serve as another attractive alternative to MSC, based on a larger number of cells extracted from tissue and a shorter doubling time than MSC, allowing for less tissue culture media use in their expansion and thus reducing the cost of production; but their various properties, such as their decreased differentiation potential, proliferation capacity and immunomodulatory properties, prefer to use MSCs [16].

Whilst various studies demonstrating the antimicrobial activity of MSCs and elucidating the mechanisms underlying this effect exist, to our best knowledge relatively little attention has been paid to investigating their impact on in vitro bacterial communication, Quorum Sensing (QS) in connection with signal molecules and the regulation of virulence factors such as biofilm production. The bacterial QS system is indispensable for biofilm formation in chronic drug-resistant infection and the manifestation of bacterial virulence activity, so it provides a major target for antibiofilm therapy [17]. Nowadays, research is focused on the development of QS antagonists as a new treatment approach for blocking communication between bacteria and for reducing virulence, therefore improving infection control. Taking into consideration that MSCs may become very promising novel therapeutic tools, and QS inhibitors in particular, the field is still unexplored and a number of experiments are required. Moreover, the regulation of bacterial communication by means of QS-dependent mechanisms through QS inhibitors that block signaling molecules provides a strategy to ‘disarm’ pathogenic microorganisms, disrupt their biofilms, paralyze their virulence and restore their susceptibility to antibiotics [18].

With regard to this issue, the main objective of the present study was to examine the effect of canine bone marrow mesenchymal stem cell-conditioned media (cBM MSC CM) on bacterial growth, biofilm-associated *Staphylococcus aureus* and acyl homoserine lactone (AHL)-dependent QS in Gram-negative bacteria.

## 2. Materials and Methods

### 2.1. Strains And Growth Conditions

*Escherichia coli (E. coli)* JM109 pSB1142: luminescence-based QS-reporter strain responding to long-chain AHLs (C10–C14) carrying the *lasR* and *lasI* promoter of *Pseudomonas aeruginosa* (*P. aeruginosa*) fused to the luxCDABE cassette from *Photorhabdus luminescens* was cultured in Brain Heart Infusion (BHI) agar (Oxoid, Basingstoke, Hampshire, UK) at 37 °C and maintained in tetracycline 20  μg/mL.

*P. aeruginosa* 45 (*lasI, lasR, rhlI, rhlR, plcH, lasB* PCR positive) clinical calf isolate was cultured in Mueller–Hinton (MH) broth (Oxoid) at 37 °C overnight.

The biofilm-associated strains *Staphylococcus aureus (S. aureus)* 14 (*hla, isdA, sdrE* PCR positive) and 11 (*hla, isdA, isdB* PCR positive) were incubated in Mannitol agar (Oxoid) at 37 °C overnight.

The standard reference strains (*Streptococcus agalacticae* CCM 6187, *Staphylococcus aureus* CCM 3953, *Escherichia coli* C 1971, *Salmonella enteritidis* CCM 4420, *Bacillus cereus* CCM 869) used for the antibacterial activity of cBM MSC CM were incubated in MH agar at 37 °C overnight.

### 2.2. Preparation of cBM MSC CM

A bone marrow sample collection from six individual healthy adult dogs (large or middle-size dog breeds, all males: Alaskan Malamute (2.0 years old), Labrador (2.3 years old), American Staffordshire Terrier (2.6 years old), Poodle (2.4 years old), Eurohound (2.7 years old), Beagle (2.4 years old)) and in vitro cultivation of cBM MSCs were performed at the University of Veterinary Medicine and Pharmacy in Košice with informed consent of the dog owners, as described in detail by Humenik et al. [19].

In order to obtain cBM MSC CM, we cultivated cBM MSCs in Dulbecco’s modified Eagle medium (DMEM) (Life Technologies, New York, NY, USA) deprived of fetal calf serum (FCS) and antibiotics. Briefly, 1.2 × 10^6^ MSCs at passage 3 were seeded in a T75 flask with DMEM containing FCS and appropriate antibiotics, namely 100 units/mL penicillin, 100 mg/mL streptomycin and 2.5 µg/mL amphotericin B (Gibco, Switzerland). After 48–72 h with a cell confluence at 80%, the medium was removed, cell monolayers were rinsed twice with phosphate buffered saline solution (PBS, Thermo Fisher Scientific, Waltham, MA, USA), and 10 mL DMEM free of FCS and antibiotics were added. The resulting medium was collected 48 h later, centrifuged twice for 10 min at 300× *g* to remove cellular debris, and frozen at −20 °C until further use in subsequent experiments. Concentrated cBM MSC CM (four-fold) were obtained using a Vacufuge plus concentrator (Eppendorf), followed by centrifugation at 7500× *g* for 40 min, applying a filter with a nominal molecular weight limit of 30,000 KDa (Amicon Ultra-0.5 30 K, Millipore, Burlington, MA, USA). Equal volumes of media (antibiotics and FCS-free DMEM) but without MSCs were handled under the same conditions and served as a negative control.

### 2.3. Anti-Bacterial Activity Test

The antimicrobial capacity of cBM MSC CM were screened against representative Gram-positive and/or Gram-negative standard reference strains and two biofilm-associated *S. aureus* strains (see Material and Methods—*Strains and Growth conditions*) using the Disc Diffusion Test (DDT), according to the EUCAST Disc Diffusion Method for Antimicrobial Susceptibility Testing [20]. Each bacterial suspension with 0.5 McFarland (1.5–3 × 10^8^ CFU/mL) was inoculated onto MH agar plates by spreading the microbial suspensions uniformly on the agar surface using cotton swabs. Sterile discs with a diameter of 10 mm were spotted with 10 μL of the four-fold concentrated cBM MSC CM or antibiotics and FCS-free DMEM (negative control–NC) and were loaded onto the plate. The plates were incubated at 37 °C, and the zones of inhibition measured after 20 h were expressed in millimeters (mm).

### 2.4. Biofilm Formation

Biofilm production was performed using SCVA, according to the previously published method by O’Toole et al. [21], with slight modifications. In brief, the biofilm-producer *S. aureus* 14 was grown on BHI agar (Oxoid), and colonies were transferred to BHI broth (Oxoid) to reach a density equivalent to 1.5–3 × 10^8^ CFU/mL. A volume of 100 μL of these bacterial suspensions, without (positive control–PC) or with the addition of 10-μL concentrated individual cBM MSC CM (containing all factors secreted by MSCs < 30 K molecular weight cutoff–experimental groups) versus antibiotic-free, FCS-free and MSC-free DMEM (negative control–NC) were inoculated into a 96-well Nunc polystyrene microtiter plate (Thermo Fisher Scientific, Roskilde, Denmark) and were incubated for 24 h at 37 °C. After incubation, sessile *S. aureus* was washed three times with PBS, fixed with methanol for 15 min, then dried and stained with 0.1% crystal violet (Sigma-Aldrich) solution for 15 min. The adhering dye was dissolved with 33% acetic acid. Eight replicates were used for each test condition/control. The results were obtained by measurement of the absorbance at λ = 550 nm in a Synergy HT Multi-Mode Microplate Reader (BioTek, Winooski, VT, USA).

### 2.5. Effect of cBM MSC CM on Cell Surface Hydrophobicity (CSH) of S. aureus

The effect of concentrated cBM MSC CM (10 μL containing all factors secreted by MSCs <30 K) on the CSH of the tested *S. aureus* was determined using the hydrocarbon (n-hexadecan) buffer two-phase system. Briefly, saline-washed *S. aureus* (1 mL, A_570_ = 0.8) without (PC) or with the addition of 10 μL of concentrated individual cBM MSC CM (experimental groups) versus antibiotics and FCS-free DMEM (NC) were incubated for 24 h. Overnight cultures in Mannitol broth at 37 °C were mixed with 1 mL of n-hexadecan (Sigma-Aldrich, Germany). After one hour of phase separation, the aqueous phase was removed, and the absorbance was measured at 570 nm using the Synergy HT Multi-Mode Microplate Reader (BioTek). CSH was then expressed as the percentage of bacterial cells adhering to n-hexadecan and was calculated using the formula:

CSH% = (A_0_ − A_1_)/A_0_ × 100 (1)

where A_0_ = initial absorbance and A_1_ = final absorbance.

### 2.6. Bioluminescence Assay (BA)

The QS reporter strain *E. coli* JM109 pSB1142 [long-chain (C10–C12) AHL biosensor] was used to measure the bioluminescence in response to incubation with individual cBM MSC CMs. The cell-free culture supernatant (CFCS) from *P. aeruginosa* 45, producing long-chain AHLs (C10–C14) and prepared according to Bermudez–Brito et al. [22], was used for bioluminescence activation in the QS-based reporter strain *E. coli* JM109 pSB1142.

Briefly, *E. coli* JM109 pSB1142 was inoculated into 2 mL of BHI broth (at a concentration of 1 McFarland) with 500 μL of *P. aeruginosa* 45 CFCS (PC). The individual concentrated cBM MSC CMs (100 μL) were added to the QS-reporter strain with CFCS of *P. aeruginosa* 45 in experimental groups, and all test tubes were incubated overnight. The 100 μL of antibiotics, FCS and MSC-free DMEM was used as NC. Eight replicates were used for each test condition/control.

The expression of the *E. coli* JM109 pSB1142 bioluminescence was observed using the Fusion FX (Vilber Lourmat) imaging system after overnight incubation at 37 °C and transferring of individual samples to white 96-well immune plates (SPL Life Sciences), after which the luminescence was measured using the Synergy HT Multi-Mode Microplate Reader (BioTek, USA). To exclude growth-dependent effects, optical density measurements were recorded in order to normalize the bioluminescence production to the cell density. The values were normalized according to bacterial growth (the absorbance was measured at λ = 550 nm-A_550_) over the same period for each of the replicates, using the same microplate reader.

### 2.7. Statistical Analysis

Statistical analyses were performed using the Statistica AXAZ software package (StatSoftCR, Czech Republic). All experiments were performed in eight replicates. *S. aureus* biofilm formation was represented as the average of absorbance values measured at λ = 550 nm (A_550_) ± standard deviation (SD) per well on 96-well Nunc polystyrene microtiter plates; bioluminescence after 24 h of growth for the bioluminescence-based reporter *E. coli* pSB1142 exposed to *P. aeruginosa* supernatant producing long-chain AHLs (C10–C14) was expressed as normalized bioluminescence treated and/or untreated with cBM MSC CM. The average values were recorded as relative light units to bacterial growth (RLU/A_550_) ± SD. A one way ANOVA and Tukey’s post hoc test were used to detect significant differences between the positive control and individual experimental groups, and a statistical significance was accepted at *p* < 0.05 level.

## 3. Results

### 3.1. Antibacterial Disc Diffusion Testing

To assess the action of nonactivated cBM MSC CM on bacterial growth, DDT was applied to selected reference-based and biofilm-associated strains.

The obtained results, when compared with control medium (NC), showed various effects evidently dependent on the bacterial type that was used. The inhibition zones varied between 7–30 mm.

Four strains (biofilm-associated *S. aureus* 14; reference-based *Escherichia coli* C1971; *Salmonella enteritidis* CCM 4420; and *Bacillus cereus* CCM 869) were resistant to all types of cBM MSC CM used (Table 1).

### 3.2. Anti-Biofilm SCVA

SCVA and biofilm-producer *S. aureus* 14 resistant to all the used cBM MSC CM was applied to examine their antibiofilm activity.

The results revealed a statistically significant biofilm inhibition in all six experimental groups (0.391 ± 0.062 in PC to the lowest 0.150 ± 0.096 in the experimental group cBM MSC CM P, and/or to the highest 0.274 ± 0.133 in the experimental group cBM MSC CM AM, given as average values of A_550_ ± SD; *p* value < 0.001; *p* value < 0.05), representing a 30–62% reduction in biofilm formation (Table 2). No differences between PC and NC was expected, which meant no biofilm inhibition in DMEM free of antibiotics, FCS and MSCs.

### 3.3. Effects of cBM MSC CM on CSH of S. aureus

CSH of *S. aureus* 14 in PC and/or NC showed 59% versus 56% adherence to n-hexadecane, respectively. In order to check the ability of cBM MSC CM to cause changes in the cell surface properties of *S. aureus* 14, the latter was exposed to n-hexadecane with the six individual types of cBM MSC CM. Type B CM produced the greatest reduction in *S. aureus* cell membrane hydrophobic properties, decreasing its hydrophobic nature by almost half when compared with that of PC, while the other five CM types had a lower effect on the bacterial surface hydrophobicity (approximately 25% reduction of adherence to hexadecane) (Table 2). The results obtained regarding CSH and/or biofilm cBM MSC CM-related changes indicate a relationship between the mentioned indices.

### 3.4. Quantification of Bioactivity of cBM MSC CM Exposed to P. aeruginosa CFCS Producing Long-Chain AHLs (C10–C14) Using Luminescence-Based Reporter E. coli JM109 pSB1142

The *E. coli* JM109 pSB1142 reporter strain was applied to quantify the residual bioactivity of cBM MSC CM-treated AHLs from *P. aeruginosa* CFCS. To exclude growth-dependent effects, optical density measurements were recorded in order to normalize the bioluminescence production to the cell density.

Table 2 shows the normalized bioluminescence results for long-chain AHLs after 24 h of incubation with the reporter strain *E. coli* JM109 pSB1142. Positive control (PC) containing the reporter strain in BHI with CFCS from *P. aeruginosa* and negative control (NC), as previously described with DMEM instead of cBM MSC CM, were used as blanks. The results demonstrated the ability of CM to modulate bacterial communication in terms of inhibiting bioluminescence in *E. coli JM109 pSB1142*, indicating AHL degradation. All six experimental cBM MSC CM groups showed a statistically significant bioluminescence inhibition with a varying force of action (11,714 ± 1362 in PC group to the lowest 7753 ± 700 in experimental cBM MSC CM AM, and/or to the highest 10,294 ± 387 in experimental cBM MSC CM L; given as average values of RLU/A_550_ ± SD; *p* value < 0.001; *p* value < 0.05). These results indicate a bioluminescence reduction in QS-related *E. coli* JM109 pSB1142 ranging from 11% to 34% (Table 2). These results suggest the capacity of cBM MSC CM to modify and degrade AHL autoinducers, thereby attenuating QS-dependent virulence in Gram negative *P. aeruginosa.* The obtained results regarding the anti-QS potential showed a differing force of action between MSCs from various individual canine samples, probably reflecting their sample-specific differences.

## 4. Discussion

The urgent need for new strategies in combating multiresistant bacterial biofilm-associated infections requires the discovery of resources other than antibiotics, and a promising strategy in the fight against pathogens appears to be to treat or control infection using QS inhibitory compounds that block the bacterial communication system, a key regulator in the production of virulence factors and biofilm formation, rather than through the direct killing of bacteria [23]. QS-therapeutic inhibitors that interfere with small molecule-controlled bacterial communication pathways could have longer functional shelf lives than second and third generation antibiotics [24], and could, moreover, reduce the evolutionary pressure to develop bacterial resistance.

The wide range of anti-QS compounds from various sources that act against QS includes halogenated furanones from marine red macroalgae *Delisea pulchra* [25,26]; N-acyl homoserine lactone acylase from *Streptomyces* species [27]; N-acyl homoserine lactonases from *Bacillus* species [28]; other unknown AHL-interfering compounds reported from various plant sources [29]; and a few marine microbes as well [30,31]. However, another attractive, insufficiently explored anti-QS supplement and cofactor to antibiotics could be MSCs possessing an immunomodulatory property that could endow them with several therapeutic and clinical applications. Recent studies have demonstrated that MSCs attenuate inflammatory responses, enhance bacterial clearance [5,32] and produce specific factors responsible for antimicrobial action. These findings suggest that MSCs, primarily safer MSC-derived secretomes (avoiding the harmful side-effects of MSC-based therapy, such as the risk of tumor formation and immunogenicity, and used in so-called cell-free stem cell therapy), could be a promising novel therapeutic modality, especially for difficult-to-treat biofilm-associated antibiotic-resistant bacterial infections. Considering that biofilm and other virulence factor productions are closely related to bacterial communication through the QS system, the question arises of whether MSC-derived secretomes might affect bacterial biofilm and signaling molecules. With regard to this, we evaluated the antibacterial, antibiofilm and anti-QS activity of cBM MSC CM that was nonactivated with bacterial preconditioning.

The main results of our study are that (a) cBM MSC CM are able to inhibit the growth of some selected bacteria (predominantly Gram-positive *Streptococcus agalacticae* CCM 6187, *Staphylococcus aureus* CCM 3953, *S. aureus* 11) and that this activity is dependent on the bacterial type that is used; (b) cBM MSC CM reduce the *S. aureus* biofilm-formation capacity without affecting bacterial growth, which points to the presence of molecules that are able to influence the bacterial-surface interaction by changing the surface area and the bioavailability of hydrophobic substrates able to modify the microbial surface hydrophobicity, thus affecting microbial adhesion and detachment from abiotic surfaces, or by inhibiting signal molecules responsible for bacterial communication and coordination in Gram-positive microbes; (c) cBM MSC CM attenuate AHL-dependent QS of the reporter strain *E. coli* JM109 pSB1142 with sample-specific suppression differences, and this inhibition of signal molecules may block the production of virulence factors, thus preventing bacterial pathogenesis and the success of the infection process.

Little is currently known about the precise mechanism underlying the antibacterial effects of MSCs. Recent data suggest that MSCs exert strong antimicrobial effects through indirect and direct mechanisms: indirectly, through their role in the host immune response against pathogens, especially in the dynamic coordination of the immune system [33,34,35,36] or by increasing the activity of phagocytes [6,32,37,38]; and directly, through the secretion of antimicrobial peptides and proteins (AMPs) [1,5,6,7,8]. Moreover, MSCs have been found to release circular membrane fragments called microvesicles (MVs), containing numerous proteins, mRNAs, microRNAs, organelles and lipids involved in cell-cell communication and the transfer of cellular material. Studies evaluating the presence of AMPs in the cargo of MVs are underlined as perspective opportunities for developing new drug delivery tools [7].

Most of the data about the antimicrobial properties of MSCs (with or without bacterial preconditioning) have been obtained from in vitro studies with bacteria. For both unstimulated and stimulated MSCs, a direct antimicrobial effect has been described.

The existing studies [1,5,39] suggest that some paracrine molecules, such as keratinocyte growth factor, antimicrobial polypeptide LL-37, or lipocalin 2, secreted by MSCs upon stimulation by bacterial preconditioning, mediate the specific antibacterial effects of MSCs. However, they admit that the antimicrobial activity of MSCs might also be associated with the other mechanisms, including the effects on phagocytosis. These variations in the antimicrobial spectrum of MSCs might be a specific response of MSCs in order to produce the most effective AMPs against a specific type of pathogen challenge or a larger quantity of unspecific molecules.

AMPs can also be active against pathogens that are resistant to conventional antibiotics, such as multidrug-resistant biofilm associated bacteria, which cause difficult-to-treat chronic diseases. Many AMPs are bactericidal as opposed to bacteriostatic, and it is unlikely that bacteria will be able to respond to these AMPs by adopting resistance strategies. These advantageous features make AMPs good candidates for drug development, although their clinical and commercial development still needs to overcome challenges, such as the route of administration, potential toxicity and stability [40]. For therapeutic use, naturally occurring AMPs may be a more practical and cost-effective substitute to synthetic ones for traditional antibiotic therapy.

To the best of our knowledge, only a few articles have, to date, studied canine MSC immune modulation [41,42,43], and no canine-derived MSC-related AMPs have been studied. Harman et al. [44] described the presence of four distinct AMPs produced by equine MSCs, namely cystatin C, elafin, lipocalin 2 and cathelicidin. With the exception of elafin, first described in the abovementioned research article, others have been documented as being produced by MSCs from other sources and species (predominantly human and murine). Similarly, canine MSCs could produce diverse antimicrobial molecules. To date, more than 3244 antimicrobial peptides from six kingdoms (364 bacteriocins/peptide antibiotics from bacteria, 5 from archaea, 8 from protists, 21 from fungi, 360 from plants and 2406 from animals, including some synthetic peptides) have been registered in the Antimicrobial Peptide Database (APD, http://aps.unmc.edu/AP/main.php; Last updated: Aug 20, 2020), including 115 that are human host defense peptides [7].

Three out of our six examined cBM MSC CM were submitted to proteomic analyses kindly provided by the Laboratoire Protéomique, Réponse Inflammatoire et Spectrométrie de Masse-PRISM, in France (the results of these proteomic analyses are shown in the research article by Humenik et al. [19], in Supp. Data 1 – List of the proteins identified). Among the proteins specified after 48 h of cBM MSC cultivation were the following: apolipoprotein B (apoB), apolipoprotein D (apoD), amyloid-β peptide (Aβ), cathepsin B and protein S100-A4, all of which can be considered to be AMPs. Apolipoproteins are generally considered to be sources of bioactive peptides known as “host defence peptides” (HDPs), on the basis of their wide range of biological activities, such as multispecies antibiofilm properties, modulation of the innate immune response, and anticancer, analgesic, antioxidant and anti-inflammatory activities. HDPs are short, cationic amphipathic peptides playing a key role in the response to infection and inflammation in all complex life forms. Gaglione et al. [45] described two novel HDPs identified in human ApoB (residues 887–922), with a broad spectrum of antimicrobial activity against both Gram-positive and Gram-negative strains. However, it has to be underlined that ApoB-derived peptides were found to be ineffective against some *S. aureus* strains and *Salmonella enteriditis,* and this observation is in agreement with previous findings indicating that most natural cationic antimicrobial peptides do not appear to be highly optimized for direct antimicrobial activity, since it is likely that multiple modestly active peptides with a concomitant immunomodulatory activity work effectively in combination and/or when induced or specifically delivered to sites of infection. The bacterial isolates treated with cBM MSC CM examined by us demonstrated various effects that were evidently dependent on the bacterial type used. Four strains (among them also *S. aureus* and *Salmonella enteritidis*) were resistant to all cBM MSC CM used by us, and these results correlate with the already-mentioned previously published facts described above for HDPs.

ApoD is an extracellular glycoprotein of the lipocalin protein family, involved in various functions such as immune response, cell proliferation regulation, chemoreception, retinoid metabolism, axon growth and proteolysis regulation [46].

Aβ is a key protein in Alzheimer’s disease pathology; however, many of the physiochemical and biological properties previously reported for Aβ are similar to those of a group of biomolecules collectively known as AMPs (see above, p. 8), which function in the innate immune system. AMPs are potent, broad-spectrum antibiotics targeting Gram-negative and Gram-positive bacteria, mycobacteria, enveloped viruses, fungi, protozoans and, in some cases, transformed or cancerous host cells, and they are also potent immunomodulators mediating cytokine release and adaptive immune responses.

The three main families of mammalian AMPs are the defensins, histatins and cathelicidins. Only one member of the cathelicidin family has been identified in humans, the LL-37 peptide, and only this one exhibits striking similarities to Aβ. Kumar et al. [47] and Soscia et al. [48] made findings showing that Aβ possessed an antimicrobial activity and might function in vivo as an AMP, thus playing a role as an effector molecule of innate immunity.

The next important contributors responding to infection as antimicrobial agents and as immune modulators (found in our CMs) are serine and cysteine proteases, for example cathepsin B [49]. Although serine proteases can kill microbes by virtue of their antimicrobial activity, unrelated to their digestive potential, these enzymes can also restrain microbial growth through the processing of microbial and host proteins. For example, they cleave virulence factors of enterobacteria or liberate host antimicrobial peptides from their precursor proteins [50].

The other, no less substantial antimicrobial superintendent detected in our CMs was one of a family of small cationic proteins, Protein S100-A4, previously reported to have proinflammatory and bactericidal properties [51].

The next important antimicrobial agents revealed by the proteomic analysis were: galectin-3, with an affinity for beta-galactosides and with an exhibition of antimicrobial activity against bacteria and fungi, as described by Feeley et al. [52]; peptides derived from C-type Lectin Domain Family 3 Member A (CLEC3A) [53]; transferrins, comprising a family of proteins that include iron-binding polypeptides and contributing to the defense against microbial infection by targeting H^+^-ATPase and interfering with H^+^ translocation, yielding a lethal effect in vitro [54]; and granulins, known as growth factors and cell communication molecules with diverse biological functions [55].

Our second task was to evaluate the antibiofilm activity of cBM MSC CM using SCVA, and our obtained results revealed a statistically significant reduction of biofilm-associated *S. aureus* 14 without the influence of bacterial growth. It may be possible that MSCs secrete molecules with the function of regulating biofilm architecture because they exhibit a broad spectrum of biofilm-inhibiting or biofilm-diffusing activities, including affecting the *S. aureus* two-component QS system encoded by the *agr* locus, which is involved in the coordination of the biofilm-formation process and bacterial virulence activities. Consequently, QS inhibitors have emerged as important promising candidates for the inhibition of biofilm formation and the expression of virulence factors through the blocking of signaling molecules. The use of these compounds alone or in combination with antibiotics may aid in the more rapid healing of chronic wounds and tissue regeneration. Another possibility for influencing bacterial-surface interaction and *S. aureus* biofilm reduction seems to be more closely related to modifications of CSH, as evidenced by our obtained results, presented in Table 2. These results indicate a relationship between the mentioned indices (biofilm production and CSH) in terms of a reduction in *S. aureus* cell membrane hydrophobic properties, decreasing its hydrophobic nature and leading to a statistically significant biofilm inhibition.

Specific cationic HDPs have recently been described as possessing a multispecies antibiofilm activity, which is independent of their effect against planktonic bacteria. The abovementioned authors, Gaglione et al. [45], pointed out that the ApoB-derived peptides examined by them displayed a significant antibiofilm activity, with, moreover, an extraordinary ability to interfere with various stages of the biofilm growth mode and simultaneously maintain bacterial viability. ApoB-derived peptides were found to be effective against biofilm formation and attachment, and were also found to strongly affect preformed biofilm. Interestingly, ApoB-derived peptide antibiofilm activity was detected even on bacterial strains that were not sensitive to direct antimicrobial activity by peptides. ApoB contains two low-density lipoprotein (LDL) receptor binding domains, namely region A (ApoB3147-3157) and region B (ApoB3359-3367), and very low-density, as well as low-density, lipoproteins play an important role in the innate immune system by interfering with the QS system, which upregulates genes required for invasive *S. aureus* infection. The mechanism of antagonism entails binding ApoB to an *S. aureus* autoinducer pheromone, preventing signaling through its receptor. For this reason, mice deficient in ApoB are more susceptible to invasive bacterial infection [56].

In our third task, we focused on the effects of cBM MSC CM with regard to the inhibition of bioluminescence in the reporter strain *E. coli* JM109 pSB1142. *P. aeruginosa* 45 produces long-chain AHLs (C10–C14) for the activation of bioluminescence in the reporter strain *E. coli* JM109 pSB1142.

We assume that cBM MSC CM contain Quorum Quenching (QQ) molecules able to degrade these parent long-chain AHLs and destroy *Las* system signaling molecules, resulting in the attenuation of AHL-dependent QS signaling. A similar statement for nonthermal plasma appears in the research article by Flynn et al. [57], who confirmed QS signaling inhibition through the chemical modification and degradation of the parent AHL molecules. QQ is a process by which QS signal molecules are enzymatically cleaved in order to inhibit their activity [58,59]. One such example is the degradation of AHL molecules by AHL lactonases and AHL acylases, which respectively cleave the homoserine lactone rings and the amide bonds of AHL molecules [60]. For example, three lactonases are present in humans: PON1, PON2 and PON3; of these, only PON2 is expressed in all tissues, and it appears to be involved in the first step of defense against bacterial infection. *P. aeruginosa* uses acyl-homoserine lactone (HSL) quorum-sensing molecules, prevalently N-(3-oxododecanoyl)-l-homoserine lactone (3O-C_12_-HSL), to regulate the expression of genes implicated in virulence and biofilm formation. It has been shown that all the human PONs can deactivate 3O-C_12_-HSL [61]. Lactonase hydrolyzes the ester bond in the homoserine lactone ring of acylated homoserine lactones. By hydrolyzing the lactone bond, lactonase prevents these signaling molecules from binding to their target transcriptional regulators, thus inhibiting quorum sensing [62]. Two other enzymes, AHL oxidase and AHL reductase, do not cleave the AHL molecule, but modify its activity. As pointed out by Teiber et al. [63], various mammal tissue and cells express a broad range of enzymes, such as carboxylesterases, amidases, acylases, proteases, oxidases and reductases, which could potentially deactivate 3O-C_12_-HSL, as well as other AHLs.

The results presented in this study demonstrate for the first time that, beyond the well-characterized antimicrobial effects of MSC CM, these conditioned media can be used to attenuate *S. aureus* biofilm production and modulate bacterial QS directly in Gram-negative bacteria. We assume that AHL molecules are altered after cBM MSC CM exposure, giving rise to AHL derivatives and, ultimately, to the complete attenuation and inhibition of signaling. This study offers the first insights into how MSC CM can rapidly alter QS signaling. At the same time, to the best of our knowledge, this is the first report on canine BM MSC CM associated with in vitro antibiofilm and anti-QS activities.

Overall, our findings suggest, first, that canine BM MSC CM inhibit the growth of some representative, predominantly Gram-positive bacteria, such as *Streptococcus agalacticae* CCM 6187, *Staphylococcus aureus* CCM 3953 and *S. aureus* 11, although some others, namely biofilm-associated *S. aureus* 14, reference-based *Escherichia coli* C 1971, *Salmonella enteritidis* CCM 4420 and *Bacillus cereus* CCM 869, remained resistant. Second, they attenuate the biofilm formation of Gram-positive *S. aureus,* and, moreover, the presented data demonstrate for the first time that cBM MSC CM exposure rapidly alters the ability of AHL to elicit QS signaling in the bacterial reporter strain *E. coli JM109 pSB1142*. These potentially vary between MSCs from various individual canine specimens and probably reflect their sample-specific differences. Furthermore, the proteomic analysis of the selected three cBM MSC CM revealed the presence of some substances of a protein character considered to be AMPs, or, more precisely, having a previously-described bactericidal effect, immunomodulation capacity and antibiofilm activity, and we assume that they are most likely responsible for the in vitro antibacterial, antibiofilm and anti-QS activities of cBM MSC CM. Although further studies will be required to give a full account of the diverse antibacterial, antibiofilm and anti-QS activity mechanisms of cBM MSC CM, these preliminary findings suggest that such conditioned media may represent an important new approach to managing biofilm-associated and QS signal molecule-dependent bacterial infections.

## 5. Conclusions

For future studies, we can conclude that cBM MSC CM, or rather their individual identified antibacterial, antibiofilm and anti-QS components, could be investigated in vivo for an evaluation of their biological activity as potent drugs in the eradication of chronic biofilm-associated drug-resistant infections in dogs, along with the therapies frequently used against them. All these observations associated with their multifunctional properties open up interesting perspectives regarding the therapeutic applications of these media.

## Figures and Tables

**Table 1:** Antibacterial DDT.

MSCs CM Samples	Inhibition Zone (mm)						
	*SA* 11	*SA* 14	*S. aureus* CCM 3953	*S. agalacticae* CCM 6187	*E. coli* C 1971	*S. enteritidis* CCM 4420	*B. cereus* CCM 869
CM AM	25	R	10	10	R	R	R
CM L	30	R	8	8	R	R	R
CM AST	20	R	7	8	R	R	R
CM P	27	R	11	12	R	R	R
CM E	24	R	10	10	R	R	R
CM B	21	R	12	12	R	R	R
NC	R	R	R	R	R	R	R

^Legend:^ R—resistant isolates; inhibition zone in mm; NC—negative control; *SA* 14—*Staphylococcus aureus* 14; *SA* 11—*Staphylococcus aureus* 11; *S. agalacticae*—*Streptococcus agalacticae*; *S. enteritidis*—*Salmonella enteritidis*; *B. cereus*–*Bacillus cereus*. Conditioned Media (CM), Alaskan Malamute (AM), Labrador (L), American Staffordshire Terrier (AST), Poodle (P), Eurohound (E), Beagle (B).

**Table 2:** Antibiofilm SCVA, anti-CSH and anti-QS activities of cBM MSC CM.

Indices	cBM MSC CM Effects		
	Anti-Biofilm ^a^ (A_550_ ± SD)	Anti-CSH ^b^ (A_570_ ± SD)	Anti-QS ^c^ (RLU/A_550_ ± SD)
PC	0.391 ± 0.062	0.331 ± 0.022 (59%)	11,714 ± 1362
NC	0.346 ± 0.099	0.349 ± 0.054 (56%)	12,253 ± 332
CM AM	0.274 ± 0.133 *	0.437 ± 0.032 (45%) ***	7753 ± 700 ***
CM L	0.197 ± 0.092 ***	0.440 ± 0.025 (45%) ***	10,294 ± 387 *
CM AST	0.246 ± 0.055 *	0.440 ± 0.052 (45%) ***	7803 ± 162 ***
CM P	0.150 ± 0.096 ***	0.450 ± 0.040 (44%) ***	9735 ± 547 ***
CM E	0.215 ± 0.047 **	0.451 ± 0.053 (44%) ***	10,230 ± 532 **
CM B	0.257 ± 0.183 **	0.556 ± 0.053 (31%) ***	8920 ± 237 ***

^a^ average values of absorbance ± SD are presented, A_550_–absorbance at λ = 550 nm; *SA 14*–*Staphylococcus aureus* 14 treated and/or untreated with conditioned media (CM) from canine bone marrow mesenchymal stem cell (cBM MSC). ^b^ average values of absorbance after adherence to n-hexadecan from eight replicate measurements ± SD are presented, A_570_–absorbance at λ = 570 nm; *SA 14* treated and/or untreated with canine bone marrow mesenchymal stem cell-conditioned media (cBM MSC CM). Optical density before adding n-hexadecane was adjusted to 0.8. The hydrophobicity was expressed as the percentage of bacterial cells adhering to n-hexadecan. ^c^ normalized bioluminescence [Relative Light Units (RLU/A_550_ ± SD)] after 24 h growth for the bioluminescence reporter *E. coli* pSB1142 exposed to *P. aeruginosa* CFCS producing long-chain AHLs (C10–C14) treated and/or untreated with cBM MSC CM. Alaskan Malamute (AM), Labrador (L), American Staffordshire Terrier (AST), Poodle (P), Eurohound €, Beagle (B). A one way ANOVA and Tukey’s post hoc test were used to detect significant differences between the positive control and individual experimental groups, and a statistical significance was accepted at * *p* value < 0.05, ** *p* value < 0.01 and *** *p* value < 0.001
